# Enhanced Treatment in Severe-Critical COVID-19 With Tocilizumab, Remdesivir, Dexamethasone: A Jordanian Cohort Study

**DOI:** 10.7759/cureus.67467

**Published:** 2024-08-22

**Authors:** Abdel-Hameed W Al-Mistarehi, Shadi El-Akawi, Khalid A Kheirallah, Ehab M Bani Ata, Khaled J Zaitoun, Ahmad B Khassawneh, Abdullah Jarrah, Hamed M Alzoubi, Sayer Al-Azzam, Reema A Karasneh, Rana B Altawalbeh, Basheer Khassawneh

**Affiliations:** 1 School of Medicine, Johns Hopkins University School of Medicine, Baltimore, USA; 2 Internal Medicine, MedStar Washington Hospital Center-Georgetown University, Washington, DC, USA; 3 Faculty of Medicine, Jordan University of Science and Technology, Irbid, JOR; 4 Faculty of Medicine, Jordan University of Science and Technology, Amman, JOR; 5 Internal Medicine, Detroit Medical Center/Sinai Grace Hospital/Wayne State University, Detroit, USA; 6 Faculty of Pharmacy, Jordan University of Science and Technology, Irbid, JOR; 7 Faculty of Medicine, Yarmouk University, Irbid, JOR

**Keywords:** treatment outcomes, covid-19 therapy, dexamethasone, remdesivir, tocilizumab

## Abstract

Background: Several medications have been proposed to manage COVID-19, with controversial data regarding their clinical benefits. We aimed to investigate the clinical efficacy of using remdesivir (RDV) with and without tocilizumab (TCZ) and standard therapy in treating severe COVID-19.

Methods: This retrospective cohort study was conducted in a Jordanian tertiary hospital (September 26th, 2020 - August 28th, 2021) and included adult COVID-19 patients requiring oxygen support. Patients were categorized into three groups based on treatment: TCZ+RDV and standard therapy; RDV and standard therapy; and standard therapy alone, which included dexamethasone, vitamins, anticoagulants, and ceftriaxone.

Results: Of 1,556 screened, 1,244 patients (mean age 62.33, 60.8% men) were included. Distribution was 106 in TCZ+RDV, 520 in RDV, and 618 in standard therapy. No significant differences were observed in age, gender, or BMI. Mortality was lowest in TCZ+RDV (32.1%), followed by RDV (40.6%) and standard therapy (47.1%) (p=0.005). Among ICU patients, TCZ+RDV showed significantly lower mortality (51.1%) compared to RDV (75%) and standard therapy (85.8%) (p<0.001). The ICU stays and invasive mandatory ventilation (IMV) durations were significantly shorter with TCZ+RDV (4.30 and 2.69 days, respectively) compared to RDV (7.61 and 4.52 days) and standard therapy (7.98 and 5.32 days) (p<0.001 for ICU stays, p=0.025 for IMV durations).

Conclusions: Combining TCZ, RDV, and dexamethasone shows promise in reducing mortality and ICU/IMV duration for severe COVID-19.

## Introduction

Since the detection of the first infected cases with a novel coronavirus, severe acute respiratory syndrome coronavirus-2 (SARS-CoV-2) in Wuhan, China, at the end of December 2019, the world has grappled with the highly transmissible coronavirus disease-2019 (COVID-19), posing an unprecedented global health challenge [1]. On March 11th, 2020, the World Health Organization (WHO) declared COVID-19 a pandemic [2]. By August 2022, nearly 590 million infected cases were confirmed worldwide, with a death toll of more than 6.5 million, according to the Johns Hopkins Coronavirus Resource Center [3]. Most COVID-19 patients experience asymptomatic or mild disease courses and recover with conservative and supportive management [4,5]. However, some develop severe symptoms, including acute respiratory distress syndrome (ARDS), severe respiratory failure, coagulopathy, and multiple organ failure necessitating hospitalization and respiratory support [6-8]. The major challenges of this disease are the highly contagious nature, with subsequently elevated rates of hospital admissions, respiratory support requirements, mortality rates, as well as the lack of definitive curative treatment yet. Consequently, strict outbreak response measures, including lockdowns, quarantine, curfew, mask-wearing, and social distancing, have been applied [9-13]. Meanwhile, exceptional efforts to develop effective medical interventions are ongoing, including efficient vaccines against SARS-CoV-2 and its variants [14] and drug therapies for infected patients [15-17]. Since the pandemic’s onset, several therapeutic agents have been proposed for managing COVID-19. They include hydroxychloroquine (HCQ), corticosteroids, antiviral drugs (favipiravir, remdesivir (RDV), nirmatrelvir/ritonavir, lopinavir/ritonavir, or molnupiravir), interleukin-6 (IL-6) receptor blockers (tocilizumab (TCZ) or sarilumab), Janus kinase (JAK) inhibitors, SARS-CoV-2-targeting monoclonal antibodies, ivermectin, convalescent plasma, lopinavir-ritonavir, and many other medications [18-21]. However, the efficacy and safety of most of these medications are still under investigation by in-vitro, animal, and clinical trials. As of August 2022, at the time of writing this article, only two medications received the United States Food and Drug Administration (USFDA) approval to be used in the treatment of hospitalized adult COVID-19 patients, which were RDV [22] and baricitinib [23]. Additionally, the USFDA issued emergency use authorization (EUA) for TCZ [24] and monoclonal antibodies [20,25]. 

Although the exact pathophysiologic mechanism(s) of COVID-19 has not been fully understood, available data illuminated the crucial role of the host immune response to viral infection in severe COVID-19 [26-28]. Infection with SARS-CoV-2 is thought to result in dysregulated immune response, myeloid cell infiltrates, activation and recruitment of monocytes, macrophages, and neutrophils, as well as the formation of neutrophil extracellular traps (NETs), complement system hyperactivation, and subsequently massive production of systemic pro-inflammatory factors, such as C-reactive protein (CRP), D-dimer, ferritin, interleukin-1 (IL-1), IL-6, and tumor necrosis factor-α (TNF-α). These immune and inflammatory mechanisms are the roots of diffuse alveolar damage, respiratory complications, vascular endothelial injury, microvascular thromboses, and multi-organ failure [26-32]. Systemic corticosteroids, particularly dexamethasone, have been suggested to mitigate these complications by modulating the exaggerated immune and inflammatory response [33]. Thus, dexamethasone is considered an effective standard treatment for hypoxic COVID-19 patients and is widely utilized in hospitalized cases [19,20,34-36].

Considering the complexity and multifaceted pathways underlying COVID-19 pathophysiology, and the lack of definitive therapeutic interventions to suppress COVID-19 morbidity and mortality rates, relying solely on a single drug may not effectively combat this deadly virus [17]. Therefore, physicians and scientists are invited to constantly explore, identify, and utilize effective combinations of the already available therapeutic COVID-19 drugs with different mechanisms of action and pharmacokinetics to control this fatal viral infection. This combination management approach would be highly applicable, especially in hospitalized severe-critical COVID-19 patients with systemic inflammation and advanced respiratory support [17-19]. However, the available evidence from clinical trials and observational studies on the efficacy of COVID-19 therapeutic agents and their combined use has been contradictory. While several studies reported no or minimal clinical benefits of COVID-19 drug combination [37-41], others indicated the added clinical values of such combination [15,17,21,42-49]. Nevertheless, the practice guidelines, adapted by the National Institutes of Health (NIH), WHO, and USFDA, recommend the combination use of multiple COVID-19 drugs, such as dexamethasone, RDV, and immunomodulatory drugs (TCZ or baricitinib), over single therapy in hospitalized COVID-19 patients with escalating oxygen needs and systemic inflammation [18-20]. Thus, this study aligns with these recommendations to determine the optimal potential pharmacologic approach for treating severe and critical cases of COVID-19. This study aimed to comprehensively evaluate the clinical efficacy of the triple combination therapy including TCZ, RDV, and standard therapy among hospitalized COVID-19 patients requiring oxygen support. We sought to address the insufficient literature regarding COVID-19 combination therapies by comparing the clinical outcomes of the combination therapy with TCZ and RDV alone, RDV monotherapy, and neither TCZ nor RDV. These regimens were evaluated in the presence of systemic corticosteroids as part of the standard COVID-19 therapy. The key clinical outcome metrics were the in-hospital and ICU mortality rates, the lengths of hospital and ICU stays, and the use of oxygen support categories.

## Materials and methods

Study design, patients, and ethical considerations

This is a retrospective cohort study conducted at the King Abdullah University Hospital (KAUH), the largest hospital in the north of Jordan, which serves a population exceeding two million and has a capacity of 750 beds [5]. It was assigned by the government as a COVID-19 referral center. The study spanned between September 26th, 2020, and August 28th, 2021 including patients hospitalized with COVID-19 and required oxygen support at any time during their hospitalization. This timeframe was not predetermined, it represents the time between the onset of the first COVID-19 peak in Jordan and the initiation of data collection [50-52]. The diagnosis of COVID-19 was confirmed by reverse-transcriptase polymerase chain reaction (PCR) detection of SARS-CoV-2 RNA in the nasopharyngeal swab. Inclusion criteria included hospitalized COVID-19 patients who were 18 years or older; had evidence of pneumonia defined as dyspnea, orthopnea, paroxysmal nocturnal dyspnea (PND), or radiologic findings of lung infiltrates; had hypoxia (SpO2 <94%) on room air; and need oxygen support of at least 6 L/min during hospitalization. On the other hand, pregnant women, mild/asymptomatic cases, and those not meeting inclusion criteria were excluded.

All procedures conducted in this study were reviewed and ethically approved by the Institutional Review Board (IRB) committee at Jordan University of Science and Technology, Irbid, Jordan (IRB number: 27/137/2021), adhering to the 1975 Helsinki Declaration, as revised in 2008, its later amendments, and comparable ethical standards. Written informed consent for participation was obtained from the patients, and the file containing the link of the patient-specific code with their name and hospital file number was locked and password protected. Thus, the patients’ information confidentiality was guaranteed, and the data analysis was conducted on the de-identified database.

Management of COVID-19 patients and study groups

Upon admission, all hospitalized COVID-19 patients have received the standard COVID-19 therapy, including dexamethasone, vitamin D3, vitamin C, paracetamol, proton pump inhibitors (PPIs), low-molecular-weight heparin (LMWH), and ceftriaxone. Systemic dexamethasone was administered intravenously (IV) at a daily dose of six mg for up to 10 days or until the patient’s discharge or death, whichever occurred first. Vitamin D3 and vitamin C were administered orally at daily doses of 5000 IU and 1000 mg, respectively. Paracetamol was administered IV or orally in divided doses up to 4 grams per 24 hours, with at least four hours between each dosage. The recommended PPI daily dosage all over the patient's hospitalization was either 40 mg of esomeprazole given by IV injection, or 30 grams of lansoprazole received orally. Also, prophylactic, or therapeutic doses of subcutaneous enoxaparin and 1 gram of IV ceftriaxone were given daily to all patients unless contraindicated. Other antibiotics, including azithromycin, levofloxacin, carbapenems, and piperacillin-tazobactam, were given as needed for superimposed nosocomial bacterial infections following the judgment of the attending physicians.

RDV was given IV with a single loading dose of 200 mg, followed by 100 mg once daily for the next five days or until hospital discharge or death, whichever occurred first. RDV therapy was extended to 10 days If the patient was mechanically ventilated and did not show clinical improvement. On October 22nd, 2020, the USFDA approved RDV use in treating COVID-19 patients requiring hospitalization and oxygenation [22]. Accordingly, the Ministry of Health (MOH) in Jordan started to import and get supplied by this medication to provide for free to severe and critical hospitalized cases requiring respiratory support with no RDV contraindications.

TCZ was administered IV with a single dose of 8 mg per kilogram of the patient's body weight, not exceeding 800 mg. A second dose could follow at least 12 hours in patients demonstrating clinical deterioration of COVID-19 signs and symptoms (e.g., increased respiratory support requirement) [53]. TCZ has not received the USFDA EUA approval for COVID-19 treatment until June 24th, 2021 [24]. Thus, TZC was given as rescue therapy for clinically worsening hypoxic adult patients having radiologic lung infiltrates and elevated inflammatory markers, with no drug contraindications. Consultation with the infectious disease team was required to ensure that patients were eligible for TCZ therapy based on COVID-19 severity criteria and drug contraindications. Moreover, written, or oral informed consent was required to obtain from the patients themselves or their legally authorized representatives. 

The use of COVID-19 medications, including dexamethasone, RDV, and TCZ, was per the management guidelines and strategies of hospitalized COVID-19 patients, published by NIH, WHO, and USFDA [18-20]. The contraindications for RDV and TCZ included: lactating women, hypersensitivity to the received drug or any of its ingredients, hepatic cirrhosis, and elevated alanine aminotransferase (ALT) or aspartate aminotransferase (AST) greater than five times the upper limit of normal (50U/L for ALT and 40U/L for AST) [38,48,54-56]. Also, RDV was used with caution in patients with severe renal impairment, defined by an estimated glomerular filtration rate (eGFR) of <30 mL/min/1.73 m2 [57]. In addition, having active bacterial, tuberculosis (TB), fungal, or viral infection other than COVID-19, platelet count of less than 100,000/mm3, absolute neutrophil count of less than 2000/mm3, and documented history of bowel diverticulitis, stomach or intestine perforation, multiple sclerosis, or demyelinating central nervous system (CNS) disorder were also identified as TCZ contraindications [38,54,56].

The enrolled patients were categorized based on the COVID-19 therapy regimen into three groups. The TCZ+RDV group compromised the patients who received the combination of TCZ plus RDV and standard COVID-19 therapy, while those treated with RDV plus standard COVID-19 therapy were classified as the RDV group. Lastly, the patients who received the standard COVID-19 therapy, neither TCZ nor RDV, were categorized as the Standard of Care (SOC) group.

Data collection and clinical evaluations

We reviewed the electronic medical charts of the enrolled patients; socio-demographic and clinical data were retrospectively collected from the patient’s electronic medical records, including age, gender, weight, height, body mass index (BMI), cigarette smoking history, and comorbidities. BMI was categorized according to the conventional WHO classification into normal weight (18.5-24.9 kg/m2), overweight (25.0-29.9 kg/m2), and obese (≥30.0 kg/m2) [58]. The comorbidities were identified based on the International Classification of Diseases (ICD) criteria [59]. The baseline clinical status of the enrolled patients at the time of admission was reviewed and abstracted from the electronic medical records, including vital signs, radiological findings, oxygen support categories, and blood laboratory values. The laboratory values were interpreted following the local reference values of KAUH. The patients were classified based on the illness severity at admission time into moderate, severe, or critical cases following the NIH classification of the clinical spectrum of SARS-CoV-2 infection [60].

The hospitalization details, including admission date, received therapies, the highest level of care, complications and serious events development, end-result of admission, discharge/death date, and oxygen support categories during hospitalization and their durations, were also obtained from the patient’s medical records. The oxygen supports were grouped into four categories: low-flow oxygen, high-flow oxygen, non-invasive ventilation (NIV), and invasive mechanical ventilation (IMV) [61]. The NIV support measures comprised continuous positive airway pressure (CPAP) and bi-level positive airway pressure (BiPAP).

Statistical analysis

The SPSS Statistics Windows software, version 25.0 (IBM Corp., Armonk, NY, USA), was used for data processing and analysis. Descriptive statistics, including frequencies and percentages, were calculated for the categorical variables. While continuous variables were presented as mean (standard deviation, SD). The univariate analyses were conducted to assess the differences between the three therapy groups using a Chi-square or Fisher’s exact test for categorical variables and one-way ANOVA for continuous variables. Regarding the primary and secondary clinical outcomes, we report the relative risk (RR) of mortality and oxygen support categories among the patients who received TCZ plus RDV and standard COVID-19 therapy compared with the patients of the RDV group. Similarly, the mean difference between the two groups of TCZ+RDV and RDV therapies was calculated and reported for the duration of hospitalization, ICU stays, and oxygen support categories. Also, their 95% confidence intervals (95% CI) and p-values were reported.

The Kaplan-Meier test was conducted to estimate the survival probability of the three therapy groups over the hospitalization duration, divided by seven-day time intervals. Also, we assessed the differences in the survival distributions between the therapy groups using the log-rank test. The estimated mean (SD) time to death after admission was calculated for each therapy arm using Kaplan-Meier analysis. Patients who were discharged alive from the hospital were censored. Cox proportional analyses were then conducted to calculate the hazard ratios (HR) for death among patients of the TCZ+RDV and RDV groups compared to patients of the SOC group.

A binary logistic regression analysis was also used to assess the predictors of in-hospital mortality among hospitalized COVID-19 patients. The dependent variable was the end-point of patient hospitalization, which was collapsed into “0 = discharge alive” and “1 = death”. Model selection using the stepwise backward approach with a cutoff p-value of 0.2 was used to select the final, most parsimonious model. The therapy regimens, age, gender, obesity, smoking status, severity on admission, radiological findings on admission, comorbidities of hypertension, diabetes mellitus (DM), ischemic heart disease (IHD), asthma, chronic obstructive pulmonary disease (COPD), chronic kidney disease (CKD), being on hemodialysis, heart failure, atrial fibrillation, cerebrovascular accident (CVA), gout, hypothyroidism, active cancer, anemia, and being immunocompromised, vasopressors use, the highest level of care (ICU or regular floor), and oxygen support categories during hospitalization were included as independent explanatory variables. The variables in the last model were checked for multicollinearity using the variance inflation factor (VIF). Adjusted odds ratios (OR) and their 95% CIs were reported. Statistical significance was considered at a p-value of ≤ 0.05.

## Results

Baseline characteristics of the patients 

**Figure 1:**
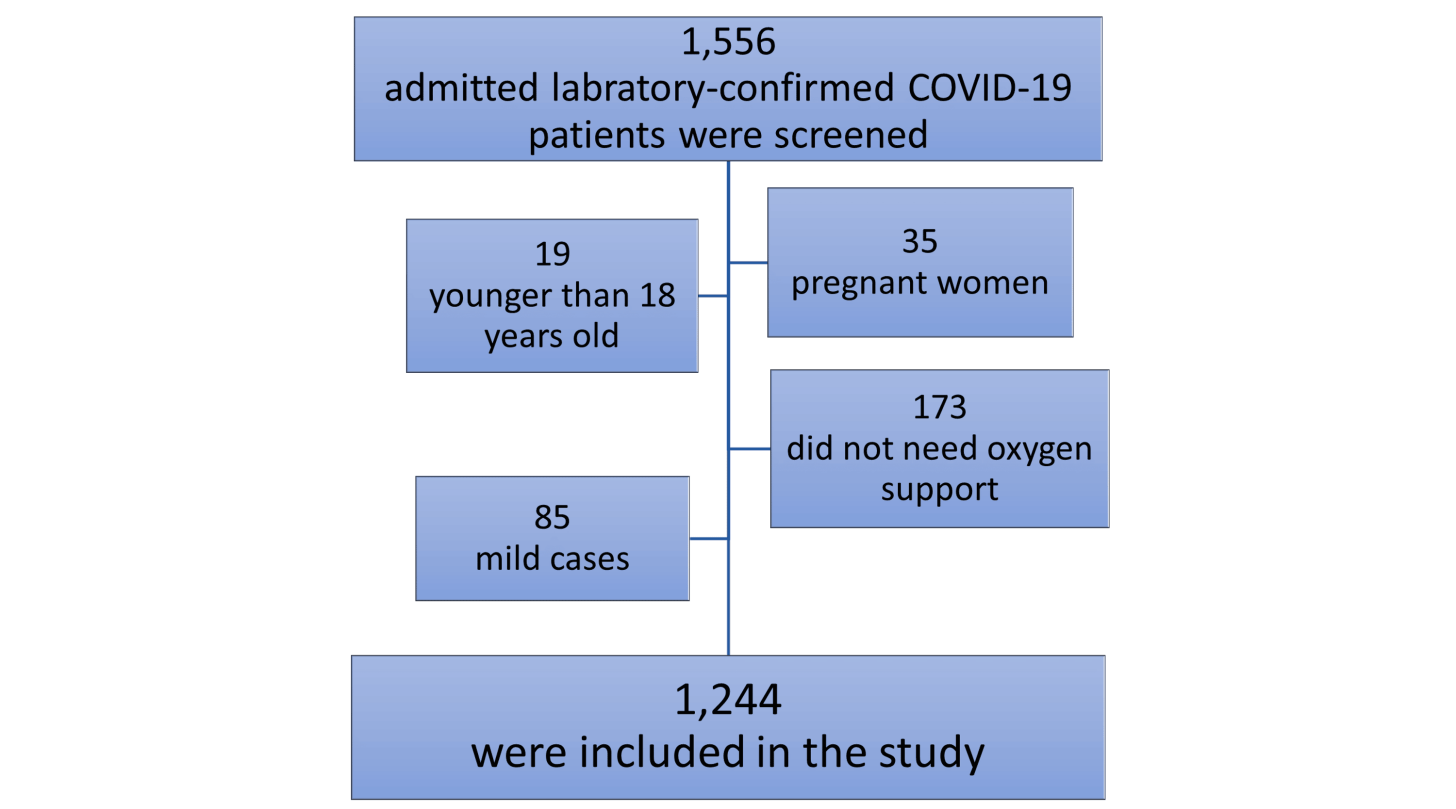
The flow chart of the study Participants.

Overall, 1,556 laboratory-confirmed SARS-CoV-2-infected consecutive patients were admitted to KAUH during the study period. Excluded patients comprised 139 younger than 18 years, pregnant women, asymptomatic, or mildly infected. Also, 173 patients were excluded as they did not require oxygen support during hospitalization. Thus, this study included 1,244 COVID-19 patients based on the inclusion and exclusion criteria (Figure 1).

The mean (SD) age of participants was 62.33 (13.83), and 756 (60.8%) were men. Moreover, their mean (SD) BMI was 30.56 (6.00), and 329 (26.6%) were ex- or current smokers. According to COVID-19 therapy, 106 (8.5%) patients received TCZ + RDV + standard COVID-19 therapy, 520 (41.8%) patients received RDV + standard COVID-19 therapy, while the SOC group consisted of 618 (49.7%) patients. The mean (SD) follow-up time was 11.53 (8.51) days, ranging from 1 to 57 days. The age, gender, and BMI were equally represented in the three therapy groups, with no statistically significant differences observed between the groups (p>0.05 for each). However, smoking status differed between the three therapy groups as the percentage of ex- and current smokers was significantly higher among the SOC cohort (30.3%) than RDV (23.3%) and TCZ+RDV groups (19.8%) (p=0.008). There were no statistically significant differences in the comorbidities between the three groups except for DM, CKD, patients on hemodialysis, and benign prostatic hyperplasia (BPH). Table 1 summarizes the baseline sociodemographic characteristics and comorbidities of the included COVID-19 hospitalized patients. 

**Table 1:** Baseline sociodemographic characteristics and comorbidities of the included COVID-19 hospitalized patients. P-value in bold indicates significance. SOC, Standard of Care; RDV, Remdesivir; TCZ, Tocilizumab; BMI, Body Mass Index; DM, Diabetes Mellitus; IHD, Ischemic Heart Disease; COPD, Chronic Obstructive Pulmonary Disease; CKD, Chronic Kidney Disease; CVA, Cerebrovascular Accident; BPH, Benign Prostatic Hyperplasia.

	Total Cohort, n=1,244 (%)	COVID-19 Treatment			P-value
		SOC group, n= 618 (49.7%)	RDV group, n= 520 (41.8%)	TCZ+RDV group, n= 106 (8.5%)	
Age (years), Mean (SD)	62.33 (13.83)	62.69 (13.48)	62.47 (13.95)	59.61 (15.11)	0.103
Age groups:					0.141
18-53 years	313 (25.2)	146 (23.6)	128 (24.6)	39 (36.8)	
54-64 years	324 (26.0)	168 (27.2)	135 (26.0)	21 (19.8)	
65-73 years	307 (24.7)	150 (24.3)	135 (26.0)	22 (20.8)	
≥ 74 years	300 (24.1)	154 (24.9)	122 (23.5)	24 (22.6)	
Gender:					0.918
Men	756 (60.8)	372 (60.2)	319 (61.3)	65 (61.3)	
Women	488 (39.2)	246 (39.8)	201 (38.7)	41 (38.7)	
BMI (kg/m2), Mean (SD), (Total n. = 1,141)	30.46 (6.00)	30.29 (6.29)	30.62 (5.65)	30.78 (5.88)	0.586
Normal, BMI 18.5-24.9	177/1,141 (15.5)	105/576 (18.2)	62/474 (13.1)	10/91 (11.0)	0.081
Overweight, BMI 25-29.9	439/1,141 (38.5)	212/576 (36.8)	185/474 (39.0)	42/91 (46.2)	
Obese, BMI ≥30	525/1,141 (46.0)	259/576 (45.0)	227/474 (47.9)	39/91 (42.9)	
Smoking status:					0.043
Non-smoker	915 (73.6)	431 (69.7)	399 (76.7)	85 (80.2)	
Ex-smoker	154 (12.4)	87 (14.1)	58 (11.2)	9 (8.5)	
Current smoker	175 (14.1)	100 (16.2)	63 (12.1)	12 (11.3)	
Any Comorbidities					
Hypertension	754 (60.6)	387 (62.6)	309 (59.4)	58 (54.7)	0.235
DM	639 (51.4)	344 (55.7)	255 (49.0)	40 (37.7)	0.001
IHD	227 (18.2)	122 (19.7)	91 (17.5)	14 (13.2)	0.232
Asthma	233 (18.7)	115 (18.6)	93 (17.9)	25 (23.6)	0.388
COPD	27 (2.2)	14 (2.3)	10 (1.9)	3 (2.8)	0.821
CKD	146 (11.7)	93 (15.0)	43 (8.3)	10 (9.4)	0.001
On hemodialysis	65 (5.2)	43 (7.0)	20 (3.8)	2 (1.9)	0.017
Heart failure	109 (8.8)	52 (8.4)	51 (9.8)	6 (5.7)	0.353
Atrial Fibrillation	47 (3.8)	21 (3.4)	23 (4.4)	3 (2.8)	0.576
CVA	84 (6.8)	46 (7.4)	35 (6.7)	3 (2.8)	0.217
Gout	103 (8.3)	50 (8.1)	44 (8.5)	9 (8.5)	0.971
Hypothyroidism	77 (6.2)	34 (5.5)	39 (7.5)	4 (3.8)	0.211
BPH among men (Total n. = 756)	123/756 (16.3)	72/372 (19.4)	47/319 (14.7)	4/65 (6.2)	0.018
Malignancy (active)	93 (7.5)	52 (8.4)	32 (6.2)	9 (8.5)	0.323
Immunocompromised	54 (4.3)	24 (3.9)	24 (4.6)	6 (5.7)	0.654

Clinical features and laboratory findings at the time of admission

The summary of the baseline vital signs, radiological findings, severity, type of oxygen support, and laboratory findings on the admission and their differences among the therapy groups are illustrated in Table 2. The prevalence rates of hypoxia were higher among the patients of the RDV and TCZ+RDV groups (90.6% and 89.6%, respectively) than in the patients assigned to SOC therapy (78.6%) (p<0.001). However, there were no statistically significant differences between the three therapy groups in fever, tachycardia, and tachypnea percentages (p>0.05 for each). The chest radiography at the time of admission showed bilateral infiltration among most participants (92.8%), with no statistically significant differences between the three groups (p=0.218). The vast majority of the participants (89.1%) were severe or critical upon admission, and 92.1% needed oxygen support at admission, with low-flow oxygenation being the most prevalent respiratory support (74.8%). Statistically significant higher percentages of RDV and TCZ+RDV groups’ patients were categorized as critical cases on admission (70.0% and 67.0%, respectively) in comparison with 54.9% among the SOC patients (p<0.001). The three therapy groups showed insignificant differences in the measurements of baseline laboratory parameters except for hemoglobin, potassium, and albumin mean values (Table 2). 

**Table 2:** Baseline clinical features and laboratory parameter findings of hospitalized COVID-19 patients at the time of admission. P-value in bold indicates significance. SOC, Standard of Care; RDV, Remdesivir; TCZ, Tocilizumab; SpO2, Oxygen Saturation using pulse oximeter; HR (bpm), Heart Rate (beats per minute); RR (bpm), Respiratory Rate (breaths per minute); BP, Blood Pressure; NIV, Non-Invasive Ventilation; CPAP, Continuous Positive Airway Pressure; BiPAP, Bi-level Positive Airway Pressure; IMV, Invasive Mechanical Ventilation; WBC, White Blood cells; Hb, Hemoglobin; MCV, Mean Corpuscular Volume; CRP, C-Reactive Protein; ESR, Erythrocyte Sedimentation Rate; LDH, Lactate Dehydrogenase; ALT, Alanine Transaminase; AST, Aspartate Aminotransferase.

	Total Cohort, n=1,244 (%)	COVID-19 Treatment			P-value
		SOC group, n= 618 (%)	RDV group, n= 520 (%)	TCZ+RDV group, n= 106 (%)	
Vital signs at admission					
Temperature (°C), mean (SD)	37.30 (0.81)	37.37 (0.83)	37.22 (0.78)	37.27 (0.82)	0.010
Fever, ≥38 °C	255 (20.5%)	139 (22.5%)	93 (17.9%)	23 (21.7%)	0.151
SpO2 (%), mean (SD)	84.98 (10.27)	84.93 (11.13)	84.65 (9.65)	86.84 (7.51)	0.134
Hypoxia, ≤92%	1052 (84.6%)	486 (78.6%)	471 (90.6%)	95 (89.6%)	<0.001
HR (bpm), mean (SD)	92.74 (16.00)	94.38 (16.66)	90.98 (15.17)	91.80 (15.18)	0.001
Tachycardia, >100 bpm	341 (27.4%)	183 (29.6%)	130 (25.0%)	28 (26.4%)	0.215
RR (bpm), mean (SD)	21.66 (2.82)	21.79 (2.89)	21.44 (2.78)	21.97 (2.51)	0.055
Tachypnea, ≥24 bpm	316 (25.4%)	167 (27.0%)	117 (22.5%)	32 (30.2%)	0.108
Systolic BP, mean (SD)	126.84 (18.94)	128.11 (19.40)	125.10 (18.84)	127.98 (15.98)	0.022
Diastolic BP, mean (SD)	74.69 (10.59)	74.99 (10.97)	74.25 (10.51)	75.10 (8.61)	0.459
Chest X-ray findings at admission, n (%)					
Normal	18 (1.4)	13 (2.1)	4 (0.8)	1 (0.9)	0.218
Unilateral infiltrates	52 (4.2)	30 (4.9)	16 (3.1)	6 (5.7)	
Bilateral infiltrates	1,155 (92.8)	563 (91.1)	494 (95.0)	98 (92.5)	
Unknown	19 (1.5)	12 (1.9)	6 (1.2)	1 (0.9)	
Severity on admission, n (%)					
Moderate	136 (10.9)	90 (14.6)	36 (6.9)	10 (9.4)	<0.001
Severe	334 (26.8)	189 (30.6)	120 (23.1)	25 (23.6)	
Critical	774 (62.2)	339 (54.9)	364 (70.0)	71 (67.0)	
Oxygen support on admission, n (%)					
Oxygen support on day 0	1,146 (92.1)	561 (90.8)	489 (94.0)	96 (90.6)	0.104
Low-flow oxygen	931 (74.8)	444 (71.8)	404 (77.7)	83 (78.3)	0.067
High-flow oxygen	53 (4.3)	28 (4.5)	24 (4.6)	1 (0.9)	
NIV (CPAP / BiPAP)	113 (9.1)	58 (9.4)	49 (9.4)	6 (5.7)	
IMV	49 (3.9)	31 (5.0)	12 (2.3)	6 (5.7)	
Clinical laboratory parameters at admission					
WBC count (× 109/L), mean (SD)	9.69 (4.90)	9.63 (4.92)	9.76 (4.91)	9.65 (4.74)	0.914
Low (<4)	62 (5.0%)	32 (5.2%)	23 (4.4%)	7 (6.6%)	0.800
Normal (4-11)	810 (65.1%)	407 (65.9%)	338 (65%)	65 (61.3%)	
High (>11)	372 (29.9%)	179 (29%)	159 (30.6%)	34 (32.1%)	
Lymphocyte count (× 109/L), mean (SD)	1.03 (1.81)	1.05 (1.79)	0.96 (1.64)	1.14 (2.48)	0.568
Lymphopenia (<1)	830 (66.7%)	400 (64.7%)	349 (67.1%)	81 (76.4%)	0.060
Hb (g/dl), mean (SD)	12.43 (2.06)	12.31 (2.09)	12.42 (1.97)	13.05 (2.17)	0.003
Anemia (<13 men & <12 women)	562 (45.2%)	298 (48.2%)	227 (43.7%)	37 (34.9%)	0.026
MCV (fL), mean (SD)	83.83 (7.47)	83.84 (7.67)	83.70 (7.39)	84.31 (6.55)	0.758
Platelets (× 109/L), mean (SD)	247.50 (106.91)	246.63 (106.20)	251.11 (109.29)	235.83 (99.66)	0.405
Low (<150)	204 (16.4%)	109 (17.6%	73 (14.0%)	22 (20.8%)	0.118
CRP (mg/L), mean (SD) (Total n. = 938)	127.73 (92.35)	133.36 (94.38)	122.11 (93.18)	116.54 (72.73)	0.111
High (>10)	896/938 (95.5%)	487/509 (95.7%)	330/346 (95.4%)	79/83 (95.2%)	0.966
ESR (mm/1hr), mean (SD) (Total n. = 1,000)	71.52 (31.69)	73.30 (31.85)	69.85 (30.57)	69.44 (35.24)	0.213
High (>22 men & >29 women)	939/1,000 (93.9%)	460/493 (93.3%)	390/413 (94.4%)	89/94 (94.7%)	0.738
D-Dimer (μg/mL), mean (SD) (Total n. = 996)	3.86 (5.25)	3.73 (5.11)	3.85 (5.32)	4.47 (5.62)	0.456
High (>1)	891/996 (89.5%)	434/477 (91.0%)	373/423 (88.2%)	84/96 (87.5%)	0.316
LDH (U/L), mean (SD) (Total n. = 949)	985.93 (461.88)	951.74 (444.61)	1017.04 (480.07)	1028.62(461.42)	0.073
High (>280)	936/949 (98.6%)	464/470 (98.7%)	386/391 (98.7%)	86/88 (97.7%)	0.746
Sodium (mmol/L), mean (SD) (Total n. = 1,132)	136.14 (5.13)	136.18 (5.23)	136.08 (5.05)	136.16 (4.93)	0.950
Low (<135)	393/1,132 (34.7%)	193/550 (35.1%)	168/478 (35.1%)	32/104 (30.8%)	0.601
Normal (135-145)	693/1,132 (61.2%)	335/550 (60.9%)	288/478 (60.3%)	70/104 (67.3%)	
High (>145)	46/1,132 (4.1%)	22/550 (4.0%)	22/478 (4.6%)	2/104 (1.9%)	
Potassium (mmol/L), mean (SD) (Total n. = 1,133)	4.54 (0.66)	4.59 (0.67)	4.50 (0.64)	4.43 (0.61)	0.017
Hypokalemia (<3.6)	64/1,133 (5.6%)	29/550 (5.3%)	28/479 (5.8%)	7/104 (6.7%)	0.386
Normal (3.6-5.2)	915/1,133 (80.8%)	435/550 (79.1%)	394/479 (82.3%)	86/104 (82.7%)	
Hyperkalemia (>5.2)	154/1,133 (13.6%)	86/550 (15.6%)	57/479 (11.9%)	11/104 (11.6%)	
Albumin (g/L), mean (SD)	33.54 (5.02)	33.99 (5.08)	32.88 (4.90)	33.72 (4.83)	0.002
Low (<35)	640 (51.4%)	317 (51.3%)	267 (53.1%)	47 (44.3%)	0.259
ALT (U/L), mean (SD)	38.15 (92.32)	38.01 (120.11)	38.16 (47.82)	38.91 (36.17)	0.996
High (>50)	169 (13.6%)	58 (9.4%)	90 (17.3%)	21 (19.8%)	<0.001
AST (U/L), mean (SD)	58.50 (174.91)	63.21 (225.95)	54.83 (100.12)	46.55 (27.31)	0.593
High (>40)	485 (39.0%)	229 (37.1%)	210 (40.4%)	46 (43.4%)	0.323
Troponin T (ng/ml), mean (SD) (Total n. = 433)	0.076 (0.358)	0.08 (0.36)	0.06 (0.37)	0.01 (0.01)	0.708
High (≥0.04)	104/433 (24.0%)	81/314 (25.8%)	19/101 (18.8%)	4/18 (22.2%)	0.354

Management, oxygenation, and complications during hospitalization 

All COVID-19 patients in this study have received systemic corticosteroids, and the vast majority (97.7%) have received at least one antibiotic, including azithromycin, levofloxacin, carbapenems, or piperacillin. About one-third of the patients needed vasopressors during their hospitalization. Table 3 shows the COVID-19 patients’ management, hospitalization site, and complications during hospitalization. There were no statistically significant differences between the three groups in the received medications except for carbapenems, piperacillin, levofloxacin, and aspirin. The percentages of patients who received carbapenems and piperacillin were higher among the TCZ+RDV group (43.4% and 64.2%, respectively) than in the other groups. On the other hand, significantly lower percentages of the TCZ+RDV patients received levofloxacin and aspirin (86.6% and 37.7%, respectively) than patients of the other groups.

**Table 3:** Treatments received, oxygenation, and significant events during hospitalization of COVID-19 patients. P-value in bold indicates significance. SOC, Standard of Care; RDV, Remdesivir; TCZ, Tocilizumab; PPIs, Proton Pump Inhibitors; LMWH, Low-Molecular-Weight Heparin; ICU, Intensive Care Unit; AKI, Acute Kidney Injury; DKA, Diabetic ketoacidosis; DVT, Deep Venous Thrombosis; PE, Pulmonary Embolism; MI, Myocardial Infarction.

	Total Cohort, n=1,244 (%)	COVID-19 Treatment			P-value
		SOC group, n= 618 (%)	RDV group, n= 520 (%)	TCZ+RDV group, n= 106 (%)	
Therapies, n (%)					
Systemic corticosteroids	1244 (100.0)	618 (100.0)	520 (100.0)	106 (100.0)	-
Azithromycin	75 (6.0)	33 (5.3)	37 (7.1)	5 (4.7)	0.382
Levofloxacin	1169 (86.8)	585 (94.7)	492 (94.6)	92 (86.8)	0.005
Carbapenems	308 (24.8)	125 (20.2)	137 (26.3)	46 (43.4)	<0.001
Piperacillin + Tazobactam	614 (49.4)	282 (45.65)	264 (50.8)	68 (64.2)	0.001
Antifungal therapy	140 (11.3)	75 (12.1)	50 (9.6)	15 (14.2)	0.250
PPIs	1,232 (99)	609 (98.5)	517 (99.4)	106 (100.0)	0.181
LMWH	1230 (98.9)	607 (98.2)	517 (99.4)	106 (100.0)	0.082
Aspirin	615 (49.4)	315 (51.0)	260 (50.0)	40 (37.7)	0.040
Other Antiplatelets (Clopidogrel, Ticagrelor, or Ticlopidine)	143 (11.5)	65 (10.5)	67 (12.9)	11 (10.4)	0.428
Statins	473 (38.0)	238 (38.5)	195 (37.5)	40 (37.7)	0.939
Insulin	703 (56.5)	354 (57.3)	289 (55.6)	60 (56.6)	0.846
Furosemide	487 (39.1)	232 (37.5)	204 (39.2)	51 (48.1)	0.120
Vasopressors	387 (31.1)	181 (29.3)	164 (31.5)	42 (39.6)	0.101
Highest level of care, n (%)					
Medical ICU	536 (43.1)	281 (45.5)	208 (40.0)	47 (44.3)	0.172
Regular medical floor	708 (56.9)	337 (54.5)	312 (60.0)	59 (55.7)	
Complications during hospitalization, n (%)					
AKI	231 (18.6)	146 (23.6)	74 (14.2)	11 (10.4)	<0.001
DKA	39 (3.1)	14 (2.3)	19 (3.7)	6 (5.7)	0.121
Pneumothorax	66 (5.3)	29 (4.7)	30 (5.8)	7 (6.6)	0.595
Emphysema	42 (3.4)	8 (1.3)	24 (4.6)	10 (9.4)	<0.001
Pleural effusion	34 (2.7)	16 (2.6)	12 (2.3)	6 (5.7)	0.148
Septic shock	143 (11.5)	76 (12.3)	56 (10.8)	11 (10.4)	0.673
Venous thromboembolism (DVT/PE)	32 (2.6)	12 (1.9)	13 (2.5)	7 (6.6)	0.020
MI	46 (3.7)	32 (5.2)	13 (2.5)	1 (0.9)	0.017
Stroke	34 (2.7)	13 (2.1)	15 (2.9)	6 (5.7)	0.112
Arrhythmias	160 (12.9)	80 (12.9)	61 (11.7)	19 (17.9)	0.221

More than half of the participants (56.9%) were on the regular floor, while 43.1% required intensive care unit (ICU) admission, with no significant differences between the therapy groups in the highest level of care (p=0.172). The most prevalent complication during hospitalization was acute kidney injury (AKI) (18.6%). The development of complications was similar among the three therapy groups except for AKI, myocardial infarction (MI), emphysema, and venous thromboembolism. The AKI and MI events were significantly more prevalent among the SOC patients than in the patients of the other groups. In contrast, emphysema and venous thromboembolism were significantly more common in the TCZ+RDV group than in other groups (Table 3).

Primary outcomes

Table 4 summarizes the clinical primary and secondary outcomes of the treated patients. Overall, 536 patients died (43.1%) in our cohort. The lowest all-cause in-hospital mortality rate was among patients assigned to receive the combination of TCZ, RDV, and standard COVID-19 therapy (32.1%), whereas it was 40.6% in the RDV group, and the highest in-hospital mortality rate was 47.1% within the SOC group (p=0.005). On day 14, the in-hospital mortality rates among the TCZ+RDV group, RDV group, and SOC group were 16.0%, 29.2%, and 34.5%, respectively (p<0.001). By day 28, 32 (30.2%) patients died in the TCZ+RDV group compared to 204 (39.2%) in the RDV group and 277 (44.8%) in the SOC group (p=0.002). Among the ICU admitted patients, 421 (78.5%) died. A considerably lower ICU mortality rate was reported among the TCZ+RDV group (51.1%) compared to the RDV group (75.0%) and those who did not take TCZ or RDV medications (85.8%) (p<0.001).

**Table 4:** Clinical outcomes in the intention-to-treat hospitalized COVID-19 patients. P-value in bold indicates significance. * Relative Risk (RR), mean difference, 95% confidence intervals (95% CI), and p-values were calculated for the TCZ+RDV group in comparison with the RDV group. SOC, Standard of Care; RDV, Remdesivir; TCZ, Tocilizumab; RR, Relative Risk; 95% CI, 95% Confidence Interval; ICU, Intensive Care Unit; NIV, Non-Invasive Ventilation; CPAP, Continuous Positive Airway Pressure; BiPAP, Bi-level Positive Airway Pressure; IMV, Invasive Mechanical Ventilation.

	Total Cohort, n=1,244 (%)	COVID-19 Treatment			P-value	RR (95% CI) or Mean difference (95% CI)*	p-value of RR or mean difference*
		SOC group, n= 618 (%)	RDV group, n= 520 (%)	TCZ+RDV group, n= 106 (%)			
Mortality rates, n (%)							
In-hospital mortality	536 (43.1)	291 (47.1)	211 (40.6)	34 (32.1)	0.005	0.790 (0.588 − 1.063)	0.102
Day 14	382 (30.7)	213 (34.5)	152 (29.2)	17 (16.0)	<0.001	0.549 (0.348 − 0.865)	0.005
Day 28	513 (41.2)	277 (44.8)	204 (39.2)	32 (30.2)	0.002	0.770 (0.565 − 1.048)	0.080
ICU mortality, n.=536	421 (78.5)	241 (85.8)	156 (75.0)	24 (51.1)	<0.001	0.681 (0.509 − 0.911)	0.001
Hospitalization durations, mean (SD)							
Total duration of hospitalization	11.53 (8.51)	10.94 (8.44)	11.37 (8.11)	15.75 (9.69)	<0.001	4.384 (2.395 − 6.372)	<0.001
For survivors, n.=708	11.53 (8.60)	10.81 (7.85)	11.19 (8.51)	16.28 (10.64)	<0.001	5.090 (2.420 − 7.760)	<0.001
For non-survivors, n=536	11.53 (8.41)	11.09 (9.07)	11.64 (7.49)	14.65 (7.27)	0.063	3.007 (0.291 − 5.724)	0.030
Duration of ICU stay	7.49 (6.25)	7.98 (6.48)	7.61 (6.29)	4.30 (3.16)	<0.001	-3.311 (-4.613 − -2.010)	<0.001
For survivors, n=73	7.08 (5.51)	10.43 (5.73)	6.27 (4.76)	3.78 (3.33)	<0.001	-2.490 (-4.952 - -0.029)	0.048
For non-survivors, n=420	7.56 (6.38)	7.69 (6.52)	7.80 (6.47)	4.79 (2.98)	0.089	-3.005 (-4.598 − -1.411)	<0.001
Oxygen support category during hospitalization, n (%)							
Low-flow oxygen	1071 (86.1)	525 (85.0)	444 (85.4)	102 (96.2)	0.007	1.127 (1.070 − 1.187)	0.002
High-flow oxygen	206 (16.6)	77 (12.5)	102 (19.6)	27 (25.5)	<0.001	1.299 (0.898 − 1.878)	0.174
Need for new high-flow oxygen	153 (12.3)	49 (7.9)	78 (15.0)	26 (24.5)	<0.001	1.635 (1.105 − 2.419)	0.016
NIV (CPAP / BiPAP)	449 (36.1)	199 (32.2)	192 (36.9)	58 (54.7)	<0.001	1.482 (1.206 − 1.822)	0.001
Need for new NIV	336 (27.0)	141 (22.8)	143 (27.5)	52 (49.1)	<0.001	1.784 (1.405 − 2.265)	<0.001
IMV	316 (25.4)	157 (25.4)	136 (26.2)	23 (21.7)	0.630	0.830 (0.562 − 1.225)	0.337
Need for new IMV	267 (21.5)	126 (20.4)	124 (23.8)	17 (16.0)	0.134	0.673 (0.424 − 1.067)	0.079
Oxygen support durations, mean (SD)							
Duration of Low-flow oxygen	7.18 (6.49)	7.07 (6.52)	7.12 (6.31)	7.93 (7.18)	0.465	0.803 (-0.595 − 2.200)	0.260
For survivors, n=681	8.49 (7.01)	8.68 (7.03)	8.19 (6.75)	8.89 (7.97)	0.616	0.702 (-1.146 − 2.550)	0.455
For non-survivors, n=385	4.84 (4.64)	4.37 (4.40)	5.20 (4.88)	5.91 (4.61)	0.093	0.707 (-1.119 − 2.532)	0.446
Duration of High-flow oxygen	4.24 (3.88)	4.42 (3.96)	3.79 (3.37)	5.44 (5.12)	0.128	1.650 (-0.470 − 3.770)	0.123
For survivors, n=126	4.55 (3.90)	4.22 (2.94)	4.29 (3.72)	5.86 (5.37)	0.219	1.572 (-0.437 − 3.581)	0.124
For non-survivors, n=80	3.76 (3.82)	4.56 (4.58)	2.60 (1.89)	3.60 (3.78)	0.093	1.000 (-1.167 − 3.167)	0.355
Duration of NIV (CPAP / BiPAP)	6.15 (5.71)	6.14 (6.16)	6.18 (5.29)	6.10 (5.55)	0.995	-0.074 (-1.652 − 1.505)	0.927
For survivors, n=95	7.45 (5.88)	7.12 (4.73)	8.56 (5.90)	6.32 (6.58)	0.273	-2.242 (-5.224 − 0.741)	0.138
For non-survivors, n=354	5.80 (5.62)	5.99 (6.34)	5.57 (4.96)	5.85 (4.18)	0.792	0.283 (-1.715 − 2.282)	0.780
Duration of IMV	4.79 (4.59)	5.32 (5.10)	4.52 (4.19)	2.69 (1.82)	0.025	-1.826 (-2.869 − -0.784)	0.001
For survivors, n=16	3.43 (2.82)	6.50 (0.84)	3.80 (3.16)	1.67 (0.58)	0.029	-2.133 (-4.461 − -0.195)	0.041
For non-survivors, n=300	4.85 (4.66)	5.32 (5.12)	4.58 (4.26)	2.85 (1.90)	0.057	-1.729 (-2.871 − -0.588)	0.004

Compared to the RDV group, the patients of the TCZ+RDV group had a significantly 45.1% reduced risk of death by day 14 (RR 0.549, 95% CI 0.348−0.865, p=0.005). Similarly, among the ICU-admitted patients, the TCZ+RDV group had a significantly 31.9% lower ICU mortality risk than patients assigned to the RDV group (RR 0.681, 95% CI 0.509−0.911, p=0.001). The risks of the overall and 28-day in-hospital mortality rates were lower in the TCZ+RDV group than in the RDV group (RR 0.790, 95% CI 0.588−1.063; and RR 0.770, 95% CI 0.565−1.048, respectively), but the difference is not statistically significant (p=0.102 and p=0.080, respectively) (Table 4).

**Figure 2:**
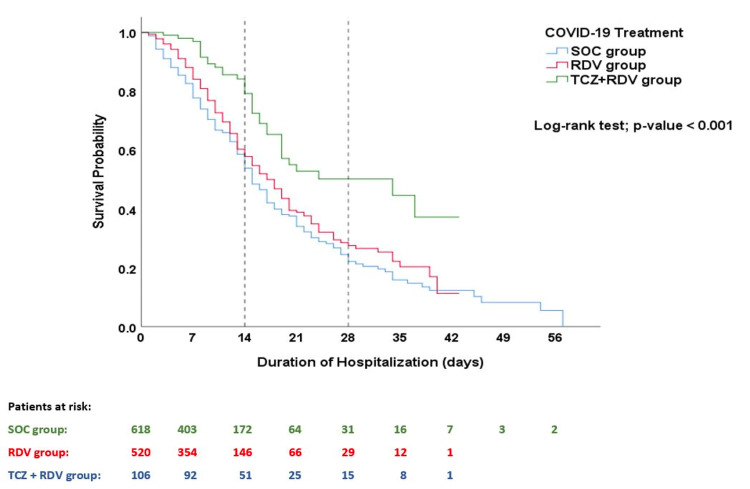
Kaplan–Meier survival analysis plot. This plot illustrates the patients’ survival probability beyond the time from hospital admission to death/discharge, with a comparison between the study groups using the log-rank test. Being alive on the day of discharge was considered a censored event. SOC, Standard of Care; RDV, Remdesivir; TCZ, Tocilizumab

The survival plots for the three therapy groups are shown in Figure 2. Survival analysis using the Kaplan-Meier test showed a significantly lower risk of mortality and a higher cumulative survival proportion among patients treated with the combination of TCZ plus RDV and standard COVID-19 therapy than in the other therapy arms (log-rank test; χ2(1) = 22.714, p<0.001) (Figure 2). The estimated mean (SD) time to death after admission was 27.96 (1.83) in the TCZ+RDV group compared to 20.67 (0.85) in the RDV group and 19.90 (0.99) in the SOC group (p<0.001). Compared to the SOC group, the HR for death in the TCZ+RDV group was 0.434 (95% CI 0.304−0.619, p<0.001), while the HR was 0.825 (95% CI 0.690−0.985, p=0.033) in the RDV group.

Secondary outcomes

Overall, the mean (SD) duration of hospitalization and ICU stay in the whole cohort were 11.53 (8.51) and 7.49 (6.25), respectively. Although the TCZ+RDV group had the longest duration of hospital stay (15.75 (9.69)) compared to the RDV group (11.37 (8.11)) and SOC group (10.94 (8.44)), the TCZ+RDV group had the shorted duration of ICU stay compared to RDV and SOC groups (4.30 (3.16) vs. 7.61 (6.29) vs. 7.98 (6.48), respectively) (p<0.001 for each comparison). Among the survivors of ICU admitted patients, the mean (SD) duration of ICU stay among the survived patients who received the combination of TCZ, RDV, and standard therapy (3.78 (3.33)) was significantly lower than the ICU stay duration among the survivors of RDV and SOC groups (6.27 (4.76) and 10.43 (5.73), respectively) (p<0.001). In contrast, among the non-survivors of ICU admitted patients, we observed no significant difference in the duration of ICU stay between the three therapy groups (p=0.089). Compared to the RDV group, the mean differences in the ICU stay duration with the TCZ+RDV group were -3.311 (95% CI -4.613 − -2.010, p<0.001), -2.490 (-4.952 - -0.029, p=0.048) among the survivors, and -3.005 (95% CI -4.598 − -1.411, p<0.001) among the dead patients (Table 4).

In the setting of oxygen therapy, most patients (86.1%) needed low-flow oxygenation during hospitalization, 16.6% required high-flow oxygenation, and 36.1% needed NIV. Patients of the TCZ+RDV group required more low-flow, high-flow, and NIV respiratory supports (96.2%, 25.5%, 54.7%; respectively) than patients of the other groups (SOC group: 85.0%, 12.5%, and 32.2%, respectively; RDV group: 85.4%, 19.6%, and 36.9%, respectively) (p=0.007, p<0.001, and p<0.001; respectively). Around one-quarter of the patients (25.4%) received IMV, with no statistically significant differences between the three therapy groups (Table 4). For patients not mechanically ventilated at baseline, the need for new IMV during hospitalization was 16.0% in those who received the combination of TCZ plus RDV and standard COVID-19 therapy, compared to 23.8% in those assigned to the RDV group (RR 0.673, 95% CI 0.424−1.067, p=0.079).

Regarding the oxygen support duration, there were no significant differences when evaluating the three therapy groups for the mean durations of low-flow oxygenation, high-flow oxygenation, and NIV, regardless of survival status (Table 4). However, the mean (SD) duration of IMV in the TCZ+RDV group (2.69 (1.82)) was significantly lower than that of IMV duration in the RDV group (4.52 (4.19)) and SOC group (5.32 (5.10)) (p=0.025). Similarly, the survivors of the TCZ+RDV group had a significantly lower mean (SD) duration of IMV use (1.67 (0.58)) than the survivors of the RDV group (3.80 (3.16)) and SOC group (6.50 (0.84)) (p=0.029).

Predictors of in-hospital mortality among the participants

Table 5 illustrates the significant predictors for in-hospital mortality using binary logistic regression analysis. The combination therapy of TCZ plus RDV and standard COVID-19 therapy was associated with the lowest odds of in-hospital mortality (adjusted OR 0.057, 95% CI 0.022−0.150, p=0.007). Also, the RDV plus standard COVID-19 therapy was associated with a double-decreased odd of death compared to SOC (adjusted OR 0.506, 95% CI 0.309−0.829, p=0.007). On the other hand, the use of vasopressors, IMV, and NIV were independent risk factors of in-hospital mortality with the highest odds ratios among the studied parameters (p<0.001 for each). Moreover, ICU admission, renal failure, CVA history, and hypertension were also identified as significant predictors of in-hospital mortality among hospitalized COVID-19 patients. Also, the current smokers had double odds of mortality compared to the nonsmokers (adjusted OR 1.919, 95%CI 1.020−3.610, p=0.043). The model showed that an age rise by one year would increase the mortality probability by 1.062 (95%CI 1.042−1.083, p<0.001).

**Table 5:** Predictors of in-hospital mortality among hospitalized COVID-19 patients. P-value in bold indicates significance. * All hospitalized COVID-19 patients enrolled in this study have received the standard COVID-19 therapy, including Dexamethasone, vitamin D3, vitamin C, paracetamol, proton pump inhibitors (PPIs), low-molecular-weight heparin (LMWH), and Ceftriaxone. OR, Odds Ratio; Ref: Reference; SOC, Standard of Care; RDV, Remdesivir; TCZ, Tocilizumab; CVA, Cerebrovascular Accident; ICU, Intensive Care Unit; NIV, Non-Invasive Ventilation; CPAP, Continuous Positive Airway Pressure; BiPAP, Bi-level Positive Airway Pressure; IMV, Invasive Mechanical Ventilation.

	Adjusted OR	95% Confidence Interval (Min. – Max.)	P-value
Study therapy groups*:			
SOC group	Ref	Ref	Ref
RDV group	0.506	0.309 − 0.829	0.007
TCZ+RDV group	0.057	0.022 − 0.150	<0.001
Age	1.062	1.042 − 1.083	<0.001
Smoking Status:			
Non-smoker	Ref	Ref	Ref
Ex-smoker	1.864	0.969 − 3.586	0.062
Current Smoker	1.919	1.020 − 3.610	0.043
Hypertension	2.615	1.567 − 4.364	<0.001
CVA History	2.691	1.020 − 7.098	0.045
On hemodialysis	10.173	2.765 − 37.423	<0.001
Immunosuppressed	2.218	0.713 − 6.901	0.169
Vasopressors	22.906	12.393 − 42.336	<0.001
ICU Admission	7.281	4.432 − 11.960	<0.001
NIV (CPAP / BiPAP)	11.765	7.045 − 19.648	<0.001
IMV	15.721	6.941 − 35.606	<0.001

## Discussion

Summary: TCZ, RDV, and standard therapy in COVID-19 treatment

Up to our knowledge, this study is the first from the Middle East and North Africa (MENA) region demonstrating clinical evidence of the superior effectiveness of TCZ, RDV, and standard therapy in combination compared to RDV plus standard therapy and standard therapy alone in treating the hospitalized COVID-19 patients requiring oxygen support. Although the need for oxygen therapy was higher among the patients of the TCZ plus RDV group than in patients of the other groups, the TCZ plus RDV group has the lowest all-cause in-hospital mortality rate. Furthermore, the 14-day and 28-day in-hospital mortality rates, as well as ICU mortality rates, were significantly lower in patients who received the combination of TCZ, RDV, and standard therapy than in the other COVID-19 therapy regimens. Although durations of low-flow, high-flow, and NIV respiratory support were not significantly different between the three therapy regimens, the TCZ plus RDV group showed significantly shorter durations of IMV use and ICU stay compared to the RDV and standard therapy groups. Among the identified predictors for in-hospital mortality rate, the combination therapy of TCZ, RDV, and standard therapy was associated with the lowest odds ratio for death.

Clinical value and outcomes of TCZ, RDV, and standard therapy

We have found that the combined use of TCZ, RDV, and standard therapy in combination for treating hospitalized COVID-19 patients requiring oxygen support was associated with in-hospital and ICU survival benefits. A few studies investigated this combination therapy and reported its clinical value in treating hospitalized COVID-19 patients [42,43,62]. On the other hand, Schneider et al. conducted a retrospective cohort study on 127 hospitalized COVID-19 patients, with 73 receiving combination therapy of TCZ, RDV, and standard therapy, while 54 patients received TCZ and standard therapy without RDV [38]. The authors found that the overall mortality rate was not statistically significant between the two study groups (17% vs. 19%, p=0.700). The lack of significance in their study could be attributed to the COVID-19 severity unmatching between the two groups, as 80% of the patients who received TCZ, RDV, and standard therapy in combination were on high-flow, NIV, IMV, or higher respiratory support levels compared to 35% of the other COVID-19 therapy arm (p<0.001) [38]. Moreover, there are concerns regarding the statistical power of their study due to the small sample size.

Respiratory support and risk factors

We observed that the need for low-flow, high-flow, and NIV respiratory support in the TCZ+RDV group is significantly higher than in the other groups. Our findings could be justified by the higher disease severity in the TCZ+RDV group than in the patients of the other therapy groups. Also, this observation could be attributed to the more prevalent development of emphysema and venous thromboembolism in the TCZ+RDV group than in other groups. A longitudinal observational study was conducted by Almaghlouth et al. on 113 COVID-19 patients, with 33 receiving TCZ plus RDV combination therapy while 80 received TCZ and methylprednisolone without RDV [39]. They reported higher odds for ventilation use among the patients who received the combination triple therapy (27.27%) compared to 11.25% in the other therapy arm (OR 2.94, 95% CI 0.12−0.95, p=0.034) [39].

Multivariate logistic regression and mortality predictors

We have established a multivariate logistic regression model to estimate the predictors for in-hospital mortality among severe and critical hospitalized COVID-19 patients. There were nine factors significantly associated with a higher mortality risk, including age, being an active smoker, hypertension, CVA history, being on hemodialysis, vasopressor use, ICU admission, and the need for NIV or IMV. These risk factors were reported elsewhere [63-67]. Schneider et al. reported that age, reduced kidney function, and higher respiratory support levels are significant risk factors for mortality, consistent with our findings [38]. A systematic review and meta-analysis of 186 studies, with 210,447 deaths among 1,304,587 COVID-19 patients, reported that smoking, obesity, and comorbidities of hypertension and diabetes as significant risk factors for mortality [65]. Our study reported the combination therapies of TCZ, RDV, and standard therapy; and RDV plus standard therapy with a lower mortality risk than standard therapy alone.

Strengths and limitations of the study

The strength points of this study include the relatively large sample size, and most vitally, this study is the first of its kind from a developing country in the MENA region evaluating the combination regimen of TCZ, RDV, and standard therapy in a longitudinal comparative design. This study compared three COVID-19 therapy regimens, including combination and monotherapy treatments. The three groups matched in the baseline demographics, most comorbidities, and laboratory and radiologic findings on admission, making the comparison valid. Also, it used inpatient record data from a tertiary hospital to reflect the reality of COVID-19 clinical management in Jordan. Most importantly, the study findings and conclusions align with the guidelines of NIH, WHO, and USFDA for managing hospitalized severe-critical COVID-19 patients.

However, this study has several limitations that require careful interpretation of the findings. First, the retrospective nature of this study and being a single-center study limit the generalization of our findings and could not prove causality-effect associations. However, the cohort design of the study comparing different COVID-19 therapy regimens could provide strong association evidence for the clinical benefits of the combination therapy. Second, the patients who received TCZ had already received RDV. Thus, we could not determine which medication was superior to the other. Third, the specific timing of TCZ administration during hospitalization and its timing in the context of the disease course and other COVID-19 drugs was not specified. This limitation could be attributed to the fact that TCZ was administered as the last option to selected patients based on clinical and contextual factors due to its high cost, unavailability in our hospital, and not being approved by the USFDA during the study period. Fourth, there were differences between the three study groups in the proportions of receiving carbapenems, piperacillin, and levofloxacin during hospitalization. Thus, we could not rule out the potential effects of these antibiotics on the outcomes in our cohort. However, previous studies comparing survivors with non-survivors among COVID-19 patients suggested no clinical benefits of antibiotic therapy on the mortality rates [7,68-70]. Finally, this study did not investigate the safety profile and the best timing for administering the COVID-19 medications. However, these aspects were not part of our study objectives. Future studies are invited to investigate and evaluate the safety issues of this combination therapy. 

## Conclusions

We concluded that the triple therapy comprising TCZ, RDV, and standard therapy demonstrates reasonable clinical efficacy in treating severe SARS-CoV-2-infected hospitalized patients, reflected by significant survival benefits, reduced all-cause in-hospital and ICU mortality rates at days 14, 28, and hospitalization end-point, as well as shorter durations of ICU stays and all-cause support. Also, the study findings elucidated the added clinical value of TCZ to the RDV and dexamethasone in achieving favorable survival outcomes by day 14 of hospitalization and for ICU admitted patients and in accomplishing quicker ICU recovery with decreased duration of IMV support.

Our study's clinical evidence-based observations correspond with NIH, WHO, and USFDA guidelines, recommending the potential combination use of COVID-19 drugs for severe-critical cases. While overall hospital stay length and respiratory support needs did not show significant improvement with TCZ, RDV, and dexamethasone therapy, this may be attributed to the severity of cases in this therapy arm and our center's TCZ administration approach. Nonetheless, patients receiving this combination therapy showed shorter and minimal use of invasive ventilation compared to other regimens. Further trials are needed to validate the efficacy and safety profile of combined TCZ, RDV, and dexamethasone therapy, and identify optimal administration timings. Physicians are encouraged to stay updated with the latest evidence on COVID-19 therapy strategies to ensure patient safety and survival.
